# Determinants of postnatal care service utilization among mothers of Mangochi district, Malawi: a community-based cross-sectional study

**DOI:** 10.1186/s12884-021-04061-4

**Published:** 2021-08-30

**Authors:** Jonas Sagawa, Allen Kabagenyi, Godwin Turyasingura, Saul Eric Mwale

**Affiliations:** 1 Community Health Department, Kamuzu University of Health Sciences, Private Bag 360, Chichiri, Blantyre 3, Malawi; 2 School of Statistics and Planning, Department of Population Studies, Makerere University, Post Office Box 7062, Kampala, Uganda; 3 Kabale University, Post office Box 317, Kabale, Uganda; 4 Biological Sciences Department, Mzuzu University, Private Bag 201, Luwinga, Mzuzu 2, Malawi

**Keywords:** Postnatal care, Cross-sectional study, Multistage sampling, Multivariable model, Mangochi district, Malawi

## Abstract

**Background:**

Postnatal care (PNC) service is a neglected yet an essential service that can reduce maternal, neonatal and infant morbidity and mortality rates in low and middle-income countries. In Malawi, maternal and infant mortality rates remain high despite numerous efforts by the government and its partners to improve maternal health service coverage across the country. This study examined the determinants of PNC utilization among mothers in Mangochi District, Malawi.

**Methods:**

A community based cross-sectional study was conducted among 600 mothers who gave birth in the past 2 years preceding January 1–31; 2016. A multistage sampling technique was employed to select respondents from nine randomly selected villages in Mangochi district. A transcribed semi-structured questionnaire was pre-tested, modified and used to collect data on socio-demographic characteristics and maternal related factors. Data was coded in EpiData version 3.1 and analysed in Stata version 12. A multivariable logistic regression adjusted for confounding factors was used to identify predictors of PNC utilization using odds ratio with 95% confidence interval and *p*-value of 0.05.

**Results:**

The study revealed that the prevalence of PNC service utilization was 84.8%. Mother’s and partner’s secondary education level and above (AOR = 2.42, CI: 1.97–6.04; AOR = 1.45, CI: 1.25–2.49), partner’s occupation in civil service and business (AOR = 3.17, CI: 1.25, 8.01; AOR =3.39, CI:1.40–8.18), household income of at least MK50, 000 (AOR = 14.41, CI: 5.90–35.16), joint decision making (AOR = 2.27, CI: 1.13, 4.57), knowledge of the available PNC services (AOR = 4.06, CI: 2.22–7.41), knowledge of at least one postpartum danger sign (AOR = 4.00, CI: 2.09, 7.50), health facility delivery of last pregnancy (AOR = 6.88, CI: 3.35, 14.14) positively associated with PNC service utilization.

**Conclusion:**

The rate of PNC service utilization among mothers was 85%. The uptake of PNC services among mothers was mainly influenced by mother and partner education level, occupation status of the partner, household income, decision making power, knowledge of available PNC services, knowledge of at least one postpartum danger signs, and place of delivery. Therefore, PNC awareness campaigns, training and economic empowerment programs targeting mothers who delivered at home with primary education background and low economic status are needed.

**Supplementary Information:**

The online version contains supplementary material available at 10.1186/s12884-021-04061-4.

## Background

Postnatal care services, as it is the case with antenatal care, labour and delivery care services, are the fundamental element of the continuum of Essential Obstetric Care (EOC) that can help in decreasing maternal, neonatal, and infant morbidity and mortality in low and middle income countries [1]. According to World Health Organization (WHO), postnatal period begins immediately after the live birth of the baby and extends up to 6 weeks (42 days) after birth and it includes an integrated package of routine maternal and neonatal care [2]. WHO recommends that a mother and her new-born child should receive postnatal care within 24 h of birth, and then at least three more times, that is to say; on day three, in the second week, and 6 weeks after the birth [2]. Appropriate PNC in the first hours and days following childbirth prevents the great majority of maternal and child morbidity and mortality [3, 4]. It is the period where the health care provider helps mothers and new-borns to establish and maintain contact with a number of health care services needed in the short and long term [3].

Each year, an estimated 529,000 women die from complications related to pregnancy and with 99% of these maternal deaths occurring in developing countries especially South Asia and sub-Saharan Africa (SSA) [4–6]. In Malawi, like many other SSA countries, maternal and child mortality rates are still high [7, 8]. Recent estimates showed that the country still experiences maternal and infant mortality rates of 439/100,000 live births and 27/1000 live births respectively [8]. Despite its importance, postnatal period is generally the most neglected in developing countries [9] including Malawi. At national level, PNC utilization within 2 weeks of delivery is currently at 42% [8]. In Mangochi district PNC utilization within 2 weeks of delivery is currently at 28% [10]. Understanding the factors related to PNC utilization is critical for countries like Malawi with an alarmingly high maternal, neonatal and infant mortality levels. Recently, studies on determinants of postnatal care utilization have been conducted in Sub-Saharan Africa, Malawi inclusive [11–26]. These studies have provided insights into the many multilevel factors that influence postnatal care utilization. Since, the determinants of PNC utilization are not the same across different cultures, different geographical locations and socio-economic status within a society [19]. This study therefore was conceptualized to examine the determinants of postnatal care service utilization among mothers in rural communities of Mangochi district, Malawi.

## Methods

### Study setting, design and sampling technique

A community based cross-sectional study was carried out from 1st to 31st January, 2016 in Mangochi district which is located in the eastern region of Malawi. The study participants were drawn from nine randomly selected villages in the district from three Traditional Authorities; Chilipa, Makanjira, and Nankumba. Six hundred women were selected using a multistage sampling technique.

### Study population and sampling procedure

Mothers who gave birth in the past 2 years preceding data collection period, with a child more than 6 weeks old, willing to participate and living in Mangochi district were included. Multistage sampling technique was used to select study subjects. Initially, all the 12 Traditional Authorities in the district were stratified into three, with four neighbouring Traditional Authorities being put in one stratum based on their geographical location. Then one Traditional Authority was selected randomly from each stratum to come up with the three Traditional Authorities to be representatives for the study. From the chosen Traditional Authorities, frame of the villages in each was drawn and three villages were chosen from each of the chosen Traditional Authorities using systematic random sampling technique. Sample size was allocated proportionally to each randomly selected village. Finally, frames of households with eligible woman were prepared for each village. Whenever more than one eligible respondent was found in the same selected household, only one respondent was chosen by lottery method.

Sample size was determined using Leslie Kish [27] sampling formula by considering 28% PNC utilization rate in Mangochi district [10], 95% level of confidence and 5% margin of error. A design effect of 1.5 and 20% non-response rate were also anticipated to obtain the final sample size of approximately 600.

### Data collection tools and procedure

The structured questionnaire was prepared using concepts in the conceptual framework and study objectives and utilized in data collection. The questionnaire was prepared in English (See Supplementary File 1) and then translated in Chichewa which is the common local language used by almost everyone in the district (See Supplementary File 2). To check its consistency, the questionnaire was back translated to English by an expert of both languages. The questionnaire was pre-tested on 10% of the population outside the study area, modified and used for data collection. Five research assistants who were fluent in the local language (Chichewa) and familiar with the local customs were recruited and used to collect the data. One research assistant who had vast experience in household surveys was assigned to supervise the data collection process. A day training was given on the objective of the study, confidentiality of information and the techniques of an interview to data collectors and supervisors by the Principal Investigator.

### Data analysis

The collected data was entered and coded in EpiData version 3.1 and analysed for both descriptive and inferential statistics using Stata Version 12 software [28]. Data analysis involved univariate, bivariate and multivariate analysis. Descriptive statistics involved generating summary statistics and frequency distribution for socio-demographic characteristics of the respondents and maternal related factors. Inferential statistics through a Pearson chi-square test was used to measure the association of socio-demographic characteristics and maternal related factors with postnatal care service utilization. A multivariable logistic regression was performed on variables such as mother’s education level, partner’s education level, occupation of the partner, age of respondents, marital status, parity, household income, decision making, knowledge of PNC services, knowledge of the postpartum danger signs and place of delivery based on either their significance in predicting PNC utilization from literature or that they were significantly associated with postnatal care service utilization at a bivariate level of analysis (*p* < 0.05) [12, 17] in order to identify the predictors of postnatal care service utilization. A multicollinearity test for individual level predictors was performed in order to control the inflationary effect of standard errors due to many predictors measuring the same variable. The variance inflation factor (VIF) and tolerance for this study were within the acceptable levels of less than or equal to 2.5 and greater than or equal to 0.4 respectively (See Supplementary file 4) for logistic regression [29]. Knowledge of PNC services, Knowledge of Postnatal danger signs and Place of delivery predictors were confounding factors since the difference in their regression coefficients between unadjusted and adjusted models was greater than 20% [30]. However, the confounding factors were maintained in the model. The model sufficiently described the data with a smaller value of Akaike’s Information Criterion (AIC) (See Supplementary File 5) and non-significant Hosmer Lemeshow test. The Odds Ratios (AOR) associated with these factors were reported as a measure of strength, together with their respective 95% Confidence Intervals and *p*-value of 0.05.

## Results

### Socio-demographic characteristics

Age, marital status, mother’s and partner’s education level, occupation status of the mother and partner, household income, distance to health facility, decision making power and family size were assessed as socio-demographic characteristics of the mothers in this study. A total of 600 women of reproductive age (15–49 years) who gave birth in the last 2 years prior to the survey were interviewed making a response rate of 100%. Mothers aged between 20 and 24 years old contributed 38.7% of the total respondents, followed by those aged 25–29 (21.8%) and 30–34 (16.0%), while the rest were either above 34 years (9.2%) or below 20 years (Table 1). With regards to marital status, slightly over eight out of 10 were in marital union, while the rest were either never married (11.7%), or divorced/widowed (4.6%) (Table 1). The majority of the mothers 64.8% had at least primary education while only 20.7% had no education at all and the same trend was seen in partners where almost 50 % had secondary education (Table 1). Regarding occupation status, at least half of the mothers 51.2% were farmers, and 20.6% were doing business, and for the partners, the majority 42.7% were farmers, 23.2% were businessmen, while 19.5% had no job at all (Table 1). About 36% of the respondents were from the poorest wealth quantile earning less than 20,000 Malawi Kwacha in a month. About 41.5% of the mothers were residing over 10 km from the health facility. Slightly above half (50.9%) of the mothers reported being involved in the decision making regarding postnatal care service utilization. Among these mothers, 27.2% were able to make the decision by themselves, while 23.7% reported of making the decision together with their husbands (Table 1). Furthermore, majority 47.0% of the respondents were from small families, with 2–4 family members, 32.2% were from medium size family and the remaining proportion were from big family size with at least six family member.

**Table 1:** Distribution of respondents by socio-demographic characteristics

Variables	Frequency (***n*** = 600)	Percentage (%)
**Age**		
15–19	86	14.3
20–24	232	38.7
25–29	131	21.8
30–34	96	16.0
35+	55	9.2
**Marital status**		
Single	70	11.7
Married	502	83.7
Divorced/Widowed	28	4.6
**Respondent’s education level**		
None	124	20.7
Primary	389	64.8
Secondary & above	87	14.5
**Partner’s education level**		
None	85	15.5
Primary	192	35.0
Secondary & above	272	49.5
**Occupation of respondent**		
None	121	20.1
House wife	48	8.0
Business	124	20.7
Farmer	307	51.2
**Occupation of partner**		
None	107	19.5
Farmer	234	42.7
Business	127	23.2
Civil servant	80	14.6
**Household Income**		
< K20,000	213	35.5
K20,000-K50,000	113	18.8
K50,000-K150,000	208	34.7
> K150,000	66	11
Distance to health facility		
0–2 Kilometers	164	27.3
3–5 Kilometers	125	20.8
6–10 Kilometers	62	10.4
> 10 Kilometers	249	41.5
**Decision making**		
Self	163	27.2
Husband	193	32.1
Joint	142	23.7
Other	102	17
**Family size**		
2–4 (Small)	282	47
5–6 (Medium)	193	32.2
> 6 (Big)	125	20.8

### Maternal related factors

Regarding the maternal related factors, the following characteristics, including parity, knowledge of postnatal care services, postnatal danger signs and place of delivery were assessed. About 41.5% of the mothers had 2–3 children and only 9.7% had more than five children by the time this study was being conducted (Table 2). Mothers were asked whether they have ever heard of available postnatal health care services, and the postnatal danger signs. 84.8% of the mothers reported having ever heard of available postnatal care services, while the rest 15.2% said they had never heard of the PNC services (Table 2). Regarding knowledge of postpartum danger signs, at least 8 out of 10 mothers reported to have ever heard of postpartum danger signs. Lastly, the highest proportion of the mothers (89.3%) delivered at the health facility while the remaining proportion had home deliveries (Table 2).

**Table 2:** Distribution of respondents by maternal related factors

Variables	Frequency (***n*** = 600)	Percentage (%)
**Parity**		
One	168	28.0
2–3	249	41.5
4–5	125	20.8
> 5	58	9.7
**Ever heard of PNC services**		
Yes	509	84.8
No	91	15.2
**Heard of postnatal danger signs**		
Yes	515	85.8
No	85	14.2
**Place of delivery**		
Home	64	10.7
Health facility	536	89.3

### Postnatal care service utilization

**Fig. 1:**
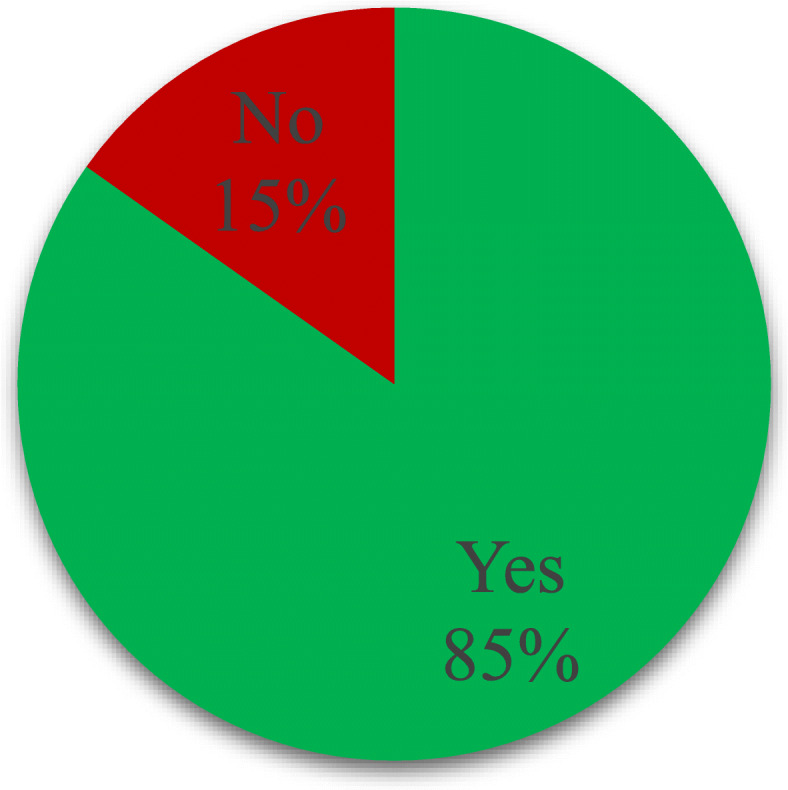
Distribution of respondents by postnatal care service utilization

Postnatal care service utilization was assessed based on binary outcome, yes or no response. In this study, a mother was considered to have utilized postnatal care services if she had attended at least one postnatal care visit within the 6 weeks post-delivery. The majority of the respondents 84.8% utilized postnatal care services while the remaining 15.2% did not utilize postnatal care services at all (Fig. 1).

### Bivariate analysis results of postnatal care service utilization

The association of postnatal care service utilization with socio-demographic characteristics and maternal related factors results are presented in Tables 3 and 4 respectively. The results show that mother’s education level, partner’s education level, occupation of the mother and partner, household income, decision making as socio-demographic characteristics were significantly associated with postnatal care service utilization (Table 3). While the following maternal related factors; knowledge of available PNC services, knowledge of postpartum danger signs and place of delivery were significantly associated with PNC service utilization (Table 4).

**Table 3:** Chi-square analysis of socio-demographic characteristics with PNC of the respondents in Mangochi, Malawi (*n* = 600)

Variables	PNC Service Utilization (%)		Chi-statistic (χ2)	***P***-values
**Age**	Yes	No	3.03	0.55
15–19	83.7	16.3		
20–24	84.0	16.0		
25–29	85.0	15.0		
30–34	83.3	16.7		
35+	92.7	7.3		
**Marital status**				
Single	81.4	18.6	1.47	0.63
Married	85.5	14.5		
Divorced/Widowed	82.1	17.9		
**Respondent’s education level**				
None	75.8	24.2	11.78	0.00
Primary	86.1	13.9		
Secondary & above	92.0	8.0		
**Partner’s education level**				
None	75.3	24.7	8.48	0.01
Primary	86.5	13.5		
Secondary & above	87.9	12.1		
**Occupation of respondent**				
None	92.6	7.4	8.65	0.03
House wife	77.1	22.9		
Business	82.3	17.7		
Farmer	84.0	16.0		
**Occupation of partner**				
None	78.5	21.5	10.67	0.01
Farmer	83.3	16.7		
Business	91.3	8.7		
Civil servant	91.2	8.8		
**Household Income**	**Yes**	**No**		
< K20,000	70.4	29.6	68.49	0.00
K20,000-K50,000	88.5	11.5		
K50,000-K150,000	96.6	3.4		
> K150,000	94.4	5.6		
**Distance to Health facility**				
0–2 Kilometers	87.2	12.8	3.44	0.33
3–5 Kilometers	84.0	16.0		
6–10 Kilometers	90.3	9.7		
> 10 Kilometers	82.3	17.7		
**Decision making**				
Self	77.9	22.1	9.21	0.03
Husband	87.1	12.9		
Joint	85.9	14.1		
Other	90.2	9.8		
**Family size**				
2–4 (Small)	83.7	16.3	1.94	0.38
5–6 (Medium)	83.9	16.1		
> 6 (Big)	88.8	11.2

**Table 4:** Chi-square analysis of maternal related factors with PNC of the respondents in Mangochi, Malawi (*n* = 600)

Variables	Postnatal Service Utilization (%)		Chi-statistic (χ2)	***P***-values
Parity	Yes	No		
One	84.5	15.5	1.72	0.63
2–3	85.5	14.5		
4–5	86.4	13.6		
> 5	79.3	20.7		
**Ever heard of PNC services**				
Yes	89.0	11.0	45.24	0.00
No	61.5	38.5		
**Heard of postpartum danger signs**				
Yes	89.3	10.7	56.89	0.00
No	57.7	42.3		
**Place of delivery**				
Home	46.9	53.1	80.23	0.00
Health facility	89.4	10.6		

### Socio-demographic predictors of postnatal care service utilization

Some variables from a bivariate analysis which were not significantly associated with PNC utilization such as mother’s age, marital status and parity but were supported by previous relevant literature were considered for inclusion in the logistic regression model. After performing a multicollinearity test as a requirement for regression analysis these variables displayed weak correlations and were included in the regression model. However, at a regression analysis level these variables still were not significantly associated with PNC service utilization (See Supplementary File 3).

The level of education of the mother and that of the partners showed a strong statistical association with postnatal care service utilization (Table 5). Mothers and their partners who attended secondary school were at least 2 times (AOR = 2.42, CI: 1.97, 6.04) and almost 1.5 times (AOR = 1.45, CI: 1.25, 2.49) respectively more likely to utilize postnatal care services than mothers and their partners who had no education at all. Similarly, respondents whose partners were in civil service were at least 3 times (AOR = 3.17, CI: 1.25, 8.01) more likely to utilize postnatal care services than mothers whose partners were not working at all (Table 5). Furthermore, mothers who were from middle income families were at least 14 times (AOR = 14.41, CI: 5.90, 35.16) more likely to utilize postnatal care services than mothers from poorest families. Lastly, involving the mother in making decision regarding postnatal care service utilization increased at least twice (AOR = 2.27, CI: 1.13, 4.57) the odds of utilizing postnatal care services among mothers of Mangochi district.

**Table 5:** Logistic regression analysis of Socio-demographic characteristics associated with PNC utilization of mothers in Mangochi, Malawi (*n* = 600)

Variables	PNC Utilization (%)		COR (CI)	AOR (CI)	*P*-values
Mother’s education level	Yes	No			
None ^R^	75.8	24.2	1.00	1.00	1.00
Primary	86.1	13.9	1.98(1.19–3.28)	1.80**(1.01–3.19)	0.005
Secondary & above	92.0	8.0	3.65(1.49–8.94)	2.31**(1.97–6.04)	0.001
Partner’s education level					
None ^R^	75.3	24.7	1.00	1.00	1.00
Primary	86.5	13.5	2.09 (1.09–4.02)	1.66 (0.88–3.13)	0.115
Secondary & above	87.9	12.1	2.38 (1.28–4.42)	1.45** (1.25–2.49)	0.004
Occupation of partner					
None ^R^	78.5	21.5	1.00	1.00	1.00
Farmer	83.3	16.7	1.37 (0.77–2.44)	2.38* (1.20–4.72)	0.039
Business	91.3	8.7	2.89 (1.32–6.34)	3.39** (1.40–8.18)	0.006
Civil servant	91.2	8.8	2.86 (1.14–7.15)	3.17* (1.25–8.01)	0.014
Household Income					
< K20,000 ^R^	70.4	29.6	1.00	1.00	1.00
K20,000-K50,000	88.5	11.5	3.23 (1.66–6.27)	2.82*** (1.40–5.70)	0.000
K50,000-K150,000	96.6	3.4	12.06 (5.08–28.65)	14.41*** (5.90–35.16)	0.000
> K150,000	94.4	5.6	7.14 (2.08–24.54)	4.63*** (1.43–15.03)	0.000
Decision making					
Self ^R^	77.9	22.1	1.00	1.00	1.00
Husband	87.1	12.9	1.90 (1.08–3.35)	1.60* (1.48–2.89)	0.017
Joint	85.9	14.1	1.73 (0.94–3.17)	2.27* (1.13–4.57)	0.031
Other	90.2	9.8	2.61 (1.22–5.58)	1.96** (1.85–4.50)	0.007

* *p* < 0.05, ** *p* < 0.01, *** *p* < 0.001, Note R = Reference category

### Maternal related predictors of postnatal care service utilization

As for maternal related factors, place of delivery showed a strong prediction for postnatal care service utilization. Mothers who gave birth with their current last born at the health facility were almost 7 times (AOR = 6.88, CI: 3.35, 14.14) more likely to utilize postnatal care services as compared to mothers who delivered at home (Table 6). Probability of postnatal care service utilization also has shown to be highly predicted by awareness of available postnatal care services. In this study, mothers who were aware of the available postnatal care services, when and where to get them were at least 4 times (AOR = 4.06, CI: 2.22, 7.41) more likely to have utilized postnatal care services than mothers who were not aware of available postnatal care services. The study also found that mothers who had knowledge of at least one postnatal obstetric danger sign were 4 times (AOR = 4.00, CI: 2.09, 7.50) more likely to utilize postnatal care services than those mothers who had never heard of postnatal danger signs at all (Table 6).

**Table 6:** Logistic regression analysis for the association between maternal-related factors and postnatal care utilization in Mangochi, Malawi (*n* = 600)

Variables	PNC Utilization (%)		COR (CI)	AOR (CI)	***P***-values
**Ever heard of PNC services**	Yes	No			
No^R^	61.5	38.5	1.00	1.00	1.00
Yes	89.0	11	5.06 (3.00–8.56)	4.06*** (2.22–7.41)	0.000
**Heard of postnatal danger signs**					
No^R^	57.7	42.3	1.00	1.00	1.00
Yes	89.3	10.7	6.14 (3.58–10.54)	4.00*** (2.09–7.50)	0.000
**Place of delivery**					
Home^R^	46.9	53.1	1.00	1.00	1.00
Health facility	89.4	10.6	9.52 (5.20–17.44)	6.88*** (3.35–14.14)	0.000

***p < 0.001. Note R = Reference category

## Discussion

The study examined the determinants of PNC service utilization among mothers in rural communities of Mangochi district, Malawi. The study revealed that 84.8% mothers utilized PNC services. The results are slightly higher than those reported by [11, 12, 20]. This may be attributed to the differences in study settings, samples size, study period and other related factors that might have led to the improvement in accessing and utilizing PNC services through time. Despite the higher percentage of PNC utilization by the mothers in the district, it’s only 5.5% of the respondents that met the set criteria of number of PNC visits by WHO and MoH, Malawi. This clearly indicates that the PNC service in the district is poorly utilized.

Socio-demographic factors such as educational level of the mothers and partners, occupation status of the partner, household level of income determined PNC service utilization. However, PNC service utilization by the mothers varied with the level of education attained by the mothers and their partners. The likelihood of utilizing PNC service was more pronounced among those with secondary level education and above than those with primary level education. The results coincide with the findings of [11–20, 31–33]. This can be attributed to the fact that education serves as a proxy for information, cognitive skills and values which in-turn promotes health seeking behavior through increased level of awareness of the available health services [11, 19]. Family disposable income obtained through an occupation of the partner or an income generating activity and joint decision making influenced PNC service utilization. Those with the ability to pay for the health care service for their wives were more likely to utilize PNC service than those without the ability to pay for the PNC service. Mothers who were involved in making decision regarding postnatal care service utilization were more likely to have utilized PNC services more than those who had to wait for either their husbands/partners or other significant others to make the decision for them [12, 21, 34–36]. reported similar findings.

Knowledge of the available PNC service, postpartum obstetric danger signs and place of delivery associated with PNC service utilization. Mothers who were aware of the available PNC service and postpartum obstetric danger signs were more likely to utilize PNC service than those who were not aware. Previous studies in developing countries also found that knowledge of postpartum danger signs and knowledge of available PNC service influences the uptake of PNC services [9, 31, 34]. This can be explained by the fact that awareness of obstetric danger sign is an important factor in triggering and motivating the mothers to seeking health care services with the aim of preventing, diagnosing and even treating the possible postnatal danger sign [19]. Additionally, knowledge is an important factor in the utilization of PNC services as it enables one to understand the services that are available, their importance, when and where to get them [21].

Mothers who delivered their last baby in the health facility utilized PNC services more when compared with those who delivered at home. This finding aligns well with the results from a study conducted in Madhya Pradesh, India [32] that reported that women who delivered at health facility utilized PNC services more than those who delivered elsewhere. This can be attributed to the fact that women who gave birth in health institutions have greater chances to get exposed to health education related to PNC services at the time of delivery and thus get access to learn about the types, benefits, and availabilities of PNC services, where and when to access them, during their stay in the health institution [19].

### Limitations of the study

Recall biasness by respondents is one of the limitation of the study as only mothers who had given birth in the past 2 years preceding data collection period were considered. Cause and effect of the predictors could not be established due to the type of research design used.

## Conclusions

PNC services are a significant constituent of safe motherhood. The study revealed that the overall utilization of PNC services among mothers in Mangochi district, Malawi was good as almost 85% of the mothers were able to utilize the services, while 15% of them had poor utilization of postnatal care services. Among the PNC service utilizers, only 5.5% utilized PNC services more than three times as recommended by WHO and Ministry of Health, Malawi. The study also established determinants of PNC utilization which should be targeted through health care awareness and sensitization campaigns on the available PNC services to enhance uptake of PNC services among mothers.

## Supplementary Information

**Additional file 1: Supplementary File 1.** Questionnaire in English.

**Additional file 2: Supplementary File 2.** Questionnaire Translated from English to Chichewa.

**Additional file 3: Supplementary File 3.** Logistic regression analysis for postnatal care service utilization on socio-demographic and maternal related factors.

**Additional file 4: Supplementary File 4.** Results of multicollinearity test for selected predictors for the multivariable model.

**Additional file 5: Supplementary File 5.** Regression diagnostics.

## Data Availability

The data sets used and analysed during the current study will be made available upon reasonable request made through the corresponding author.

## Abbreviations

AOR: Adjusted Odds Ratio
COR: Crude Odds Ratio
CI: Confidence Interval
EOC: Essential Obstetric Care
IEC: Information Education and Communication
PNC: Postnatal Care
USAID: United States Agency for International Development
WHO: World Health Organization
Coef.: Coefficient
MOH: Ministry of Health
