# Supplementary material for: Determinants of postnatal care service utilization among mothers of Mangochi district, Malawi: a community-based cross-sectional study
Source: BMC Pregnancy Childbirth. 2021 Aug 30;21:591. doi: 10.1186/s12884-021-04061-4 (PMC8406845; doi:10.1186/s12884-021-04061-4)
Supplement: Supplementary file 1 — Additional file 1: Supplementary File 1. Questionnaire in English. [file 12884_2021_4061_MOESM1_ESM.docx]

# Appendix 5: Questionnaire *Confidential*

**TITLE: DETERMINANTS OF POSTNATAL CARE UTILIZATION AMONG MOTHERS IN MANGOCHI DISTRICT, MALAWI**

**Invitation:** You are being invited to voluntarily participate in this study on determinants of postnatal care utilization among mothers in Mangochi District, Malawi. Postnatal care is one of the Maternal and Child Health care interventions that can help in preventing Maternal, Neonatal and Infant mortality rates which are still high in our country. Therefore, it is important that we find some of the factors that affect its utilization in order to help the health care providers and other stakeholders including the government to develop ways of improving its uptake. You will be required to answer the questions below regarding the topic. You will also be requested to allow us to check in the health passport book for you and your baby to collect some information. Your participation is highly valued.

**Instruction to the researcher/ research assistant**: Circle the participants’ responses, write code of the participant’s response appropriately in the box provided and where applicable write the required responses in the spaces provided.

**HOUSEHOLD No**:

**DATE**  / / /

**VILLAGE CODE**:

**INTERVIEWER’S INITIALS:**

**SECTION A: SOCIO-DEMOGRPHIC INFORMATION**

| **No** | | **QUESTIONS AND FILTERS** | | **CODING CATEGORIES** | **SKIP** |
| --- | --- | --- | --- | --- | --- |
| 001 | | How old are you? | | Age in complete years |  |
| 002 | | When were you born? | | / / / / |  |
| 003 | | What is your marital status? | | 1. Single 2. Married 3. Divorced 4. Widowed |  |
| 004 | | What is your religious affiliation? | | 1. Roman Catholic 2. CCAP 3. SDA 4. Islam   99. Other (specify)………………….. |  |
| 005 | | What is your ethnicity? | | 1. Chewa 2. Lhomwe 3. Yao 4. Tumbuka 5. Sena   99. Others (specify)…………………. |  |
| 006 | | How many pregnancies have you had? | | 1. One 2. 2-3 3. 4-5 4. >5 |  |
| 007 | | How many live births have you had? | | 1. One 2. 2-3 3. 4-5 4. >5 |  |
| **SECTION B: SOCIO-ECONOMIC INFORMATION** | | | | | |
| 008 | | What is your highest level of education? | | 1. None 2. Primary 3. Secondary 4. Tertiary |  |
| 009 | | What is the education level of your partner/husband? | | 1. None  2. Primary  3 Secondary  4. Tertiary |  |
| 010 | | What is your occupation status? | | 1. None 2. Student 3. House wife 4. Maid servant 5. Farmer 6. Civil servant 7. Others (specify)…………………… |  |
| 011 | | What is the occupation status of your husband/partner? | | 1. None  2. Student  3. Maid servant  4. Farmer   1. Business 2. Civil servant   99. Others (specify)…………………… |  |
| 012 | | How much on average do you earn per month as a family? | | 1. <MK20,000 2. MK20, 000 to MK50,000 3. MK50,000 to MK100,000 4. MK100,000 toMK150,000 5. MK150,000 to MK200,000 6. MK200,000 to MK250,000 7. >MK250,000 |  |
| 013 | | How long is it from your home to the nearest health facility? | | 1. 0-2 km 2. 3-5 km 3. 6-10 km 4. ≥ 11km |  |
|  | | **SECTION C: SOCIO-CULTURAL INFORMATION** | | | |
| 014 | | Who is the head of this household? | | 1. Husband 2. Myself   99. Others (specify)…………………… |  |
| 015 | | Who makes final decision regarding health care utilization? | | 1. Self 2. Husband 3. Both my husband and my self   99. Others (specify)………………… |  |
| 016 | | How many people live in this house? | | 1. 2-4 2. 5-6 3. ≥7 |  |
|  | | **SECTION D: POSTNATAL CARE KNOWLEDGE** | | | |
| 017 | | Have you ever heard of  Postnatal care? | | 1. Yes 2. No | If 2 skip to 020 |
| 018 | | Where did you hear about it from? | | 1. Friends 2. Health worker 3. Radio 4. Relatives   99. Others (specify)………………… |  |
| 019 | | What services are offered at postnatal clinic? | | 1. Child immunization 2. Family planning 3. Treatment of illnesses 4. Growth monitoring 5. Assessment of mother and baby   99. Others (specify)………………… |  |
| 020 | | How many times should a mother and the baby have to attend Postnatal care clinic | | 1. Once 2. 2-3 times 3. ≥4 |  |
|  | **SECTION E: KNOWLEDGE OF PNC DANGER SIGNS** | | | | |
| 021 | Have you ever heard of PNC danger signs? | | 1. Yes 2. No | | If 2 skip to 024 |
| 022 | What postnatal danger signs do you know? | | 1. Bleeding 2. Convulsions 3. Lower Abdominal Pains 4. Severe headache 5. Dizziness 6. Fever 7. Failing to breastfeed 8. Umbilical cord infection   99. Others (specify)……………………… | |  |
| 023 | **SECTION F: POSTNATAL CARE UTILZATION** | | | |  |
|  | Where did you give birth to for your baby? | | 1. Home 2. Health facility | |  |
| 024 | Did you attend PNC? | | 1. Yes 2. No | | If 2 skip to 034 |
| 025 | How many postnatal visits did you have? | | 1. None  2. 1-3  3. ≥4 | |  |
| 026 | Who attended to you during the PNC visits? | | 1. Nurse/Midwife 2. Clinician/Doctor 3. Student 4. Support staff | |  |
| 027 | What services were you seeking at postnatal clinic? (more than one item can be ticked) | | 1. To seek treatment 2. Child’s immunization 3. Family planning 4. Check-up for both mother and baby   99. Others (specify)…………………………. | |  |
| 028 | What did you like most during the postnatal visits? | | 1. Cleanliness 2. Friendly health personnel 3. Given adequate treatment 4. Good health education 5. Attended fast   99. Others (specify)…………………………... | |  |
| 029 | What did you not like most during the postnatal visits? | | 1. Long waiting time 2. Impolite by health workers 3. Attended by student 4. Clients not served on first come basis   99. Others (specify)…………………………... | |  |
| 030 | Who influenced you to utilize postnatal care services? | | 1. Mother 2. Friends 3. Neighbour 4. Church members   99. Others (specify)…………………………… | |  |
| 031 | How did you perceive the quality of PNC services? | | 1. Excellent 2. Very good 3. Poor 4. Very poor   99. Others (specify)……………………………. | |  |
| 032 | What was your perception of the health care provider who attended to you? | | 1. Friendly 2. Rude 3. Selfish   99. Others (specify)………………………… | |  |
| 033 | How long did you wait before you were attended? | | 1. <1 hour 2. 1-2 hours 3. 3≥ hour | |  |
| 034 | What was the most reason that made you not to utilize PNC services? | | 1. No/little knowledge 2. Being healthy 3. Being busy 4. Far from home 5. Long waiting time   99. Others (specify)……………………….. | |  |
| 035 | What do you think should be done to improve PNC utilization in Mangochi  District? (More than one can be ticked) | | 1. Reduce waiting time 2. Health workers need to be polite 3. Supervise students 4. Employ more health workers 5. Introduce shifts to attend clients on lunchtime   99. Others (specify)………………… ……….. | |  |

*Thank you very much for your time and responses*
