# Supplementary material for: Determinants of postnatal care service utilization among mothers of Mangochi district, Malawi: a community-based cross-sectional study
Source: BMC Pregnancy Childbirth. 2021 Aug 30;21:591. doi: 10.1186/s12884-021-04061-4 (PMC8406845; doi:10.1186/s12884-021-04061-4)
Supplement: Supplementary file 2 — Additional file 2: Supplementary File 2. Questionnaire Translated from English to Chichewa. [file 12884_2021_4061_MOESM2_ESM.docx]

**MUTU: ZINTHU ZOMWE ZIMATHANDIDZILA KAPENA KULEPHERETSA AMAYI OMWE ABELEKA KUPITA KUKALANDIRA CHITHANDIZO CHOMWE MAYI YEMWE WABELEKA AMAYENERA KULANDIRA M’BOMA LA MANGOCHI KU MALAWI**

**Kupempha:** Muli kupemphedwa kuti mutengepo gawo mu kafukufuku yemwe mutu wake ndi kufuna kudziwa zinthu zomwe zimathandidzila kapena kulepheretsa amayi omwe abeleka kupita kukalandira chithandizo chomwe mayi wina aliyense yemwe wabeleka amayenera kulandira m’boma lino la Mangochi. Chithandizo chomwe amayi omwe abeleka amayenera kulandira akabeleka ndi chofunikira kwambiri chifukwa chimathandidza kuchepetsa imfa amayi ndi ana. Kotero, ndikofunika kwambiri kuti tidziwe zifukwa zomwe zimathandidzila kapena kulepheretsa amayi omwe abeleka kupita kukalandira chithandizo chomwe mayi wina aliyense yemwe wabeleka amayenera kulandira m’boma lino la Mangochi. Mudzafunsidwa kuyankha mafunso omwe adzakufunsani wopangitsa kafukufukuyu okhuzana ndi mutu womwe tikufufudzawu. Mudzapemphedwanso kuti wommwe akupanga kafukufukuwa aone nawo buku la ku chipatala lanu ndi la mwana wanu. Kutenga nawo kwanu mbali pa kafukufuku ameneyu nkotamandika kwambiri.

**Malangizo kwa wopangitsa kafukufuku**: Lembani yankho lomwe wotenga mbali mu kafukufukuyu apereka mu bokosi lomwe la perekedwa pa funso lililonse, kapenanso kulemba yankho lonse m’mipata yaperekedwayo ngati nkoyenera kutelo.

**Nambala ya khomo**:

**Tsiku:**  / / /

**Kodi ya mudzi**:

**Ma inisho a wopangitsa**

**kafukufuku:**

**GAWO LOYAMBA: MBIRI YANU**

| **No** | | **QUESTIONS AND FILTERS** | | **CODING CATEGORIES** | **SKIP** |
| --- | --- | --- | --- | --- | --- |
| 001 | | Muli ndi zaka zingati? | | Zaka zonse |  |
| 002 | | Munabadwa liti? | | / / / / |  |
| 003 | | Zokhuza banja? | | 1. Wosakwatiwa 2. Wokwatiwa 3. Tinasiyana 4. Wamasiye |  |
| 004 | | Ndinu a chipembezo chanji? | | 1. Katolika 2. CCAP 3. SDA 4. Nsilamu   99. Zina (Nenani)………………….. |  |
| 005 | | Ndinu a ntundu wanji wa anthu? | | 1. Chewa 2. Lhomwe 3. Yao 4. Tumbuka 5. Sena   99. Zina (Nenani)…………………. |  |
| 006 | | Mwakhalapo ndi mimba zingati? | | 1. Imodzi 2. 2-3 3. 4-5 4. Kupyola zisanu |  |
| 007 | | Mwabelekapo ana angati a moyo? | | 1. M’modzi 2. 2-3 3. 4-5 4. Kupyola asanu |  |
|  | | **GAWO LACHIWIRI: ZA MOYO WA TSIKU NDI TSIKU** | | | |
| 008 | | Sukulu munalekedza pati? | | 1. Sindinaphunzire 2. Pulaimale 3. Sekondale 4. Koleji |  |
| 009 | | Mwamuna/bwenzi wanu sukulu analekedza pati? | | 1. Sanaphunzire  2. Pulaimale  3 Sekondale  4. Koleji |  |
| 010 | | Mumagwira ntchito yanji? | | 1. Sindigwira ntchito 2. Wophunzira 3. Mayi wapakhomo 4. Ya m’nyumba 5. Bizinesi 6. Ulimi 7. Ya boma   99. Zina (nenani)…………………… |  |
| 011 | | Mwamuna/bwenzi wanu amagwira ntchito yanji? | | 1. Sagwira ntchito  2. Ali pa sukulu  3. ya m’nyumba  4. Ulimi   1. Bizinesi 2. Ya boma   99. Zina (nenani)…………………… |  |
| 012 | | Mumapedza ndalama zochuluka bwanji pa mwezi ngati banja? | | 1. <MK20,000 2. MK20, 000 to MK50,000 3. MK50,000 to MK100,000 4. MK100,000 toMK150,000 5. MK150,000 to MK200,000 6. MK200,000 to MK250,000 7. >MK250,000 |  |
| 013 | | Mumayenda ntunda wautali bwanji kuchokera kunyumba kwanu kukafika ku chipatala chapafupi? | | 1. 0-2 km 2. 3-5 km 3. 6-10 km 4. ≥ 11km |  |
|  | | **GAWO LACHITATU: ZA CHIKHALIDWE CHANU** | | | |
| 014 | | Mutu wa banja lino ndi ndani? | | 1. Amuna anga 2. Ineyo   99. Ena (nenani)…………………… |  |
| 015 | | Amapanga chiganizo chopita kukalandira chithandizo cha za umoyo ndi ndani? | | 1. Ndekha 2. Amuna anga 3. Ine ndi amuna anga limodzi   99. Zina (Nenani)………………… |  |
| 016 | | Ndi anthu angati omwe amakhala m’nyumba muno? | | 1. 2-4 2. 5-6 3. Wopyola asanu ndi m’modzi |  |
|  | | **GAWO LACHINAYI: ZOMWE MUKUDZIWA PA CHISAMALIRO CHOPEREKEDWA KWA MAYI AKABEREKA** | | | |
| 017 | | Munamvapo za chisamaliro chopelekedwa kwa mayi akabereka? | | 1. Eya 2. Ayi | Ngati ayi pitani ku funso 020 |
| 018 | | Munazimvera kuti? | | 1. Anzanga 2. A za umoyo 3. Pa wailesi 4. Achibale   99. Ena (Nenani)………………… |  |
| 019 | | Ndi chisamaliro chanji chomwe chimapelekedwa ku sikelelo ya amayi omwe abeleka? | | 1. Katemera wa mwana 2. Njira zakulera 3. Chithandizo cha matenda 4. Kuona momwe mwana   akukukulira  99. Zina (Nenani)………………… |  |
| 020 | | Ndi maulendo angati omwe mayi ndi mwana amayenera kupita ku sikelelo ya amayi omwe abeleka? | | 1. Kamodzi 2. Kawiri kapena katatu 3. Kupyola katatu |  |
|  | **GAWO LACHISANU: ZOMWE MUKUDZIWA PA ZIZINDIKIRO ZOOPSA ZOMWE ZIMADZA MAYI AKABEREKA** | | | | |
| 021 | Munanvapo za zizindikiro zoopsa zomwe zimadza mayi akabereka? | | 1. Eya 2. Ayi | | Ngati ndi ayi pitani funso 024 |
| 022 | Ndi zizindikiro zoopsa zanji zomwe mukudziwa zomwe zimadza mayi akabereka? | | 1. kutaya magazi 2. kukomoka 3. kuwawa kwambiri kwa m’mimba 4. kuwawa kwambiri kwa mutu 5. Chizungulire 6. Kutentha kwa thupi 7. Kulephera kuyamwitsa 8. kufiila kapena mafinya   panchombo pa mwana  99. Zina (Nenani)……………………… | |  |
| 023 | **GAWO LA CHISANU NDI CHIMODZI: ZA KULANDIRA CHITHANDIZO KUTSATIRA KUBELEKA** | | | |  |
|  | Munabelekera kuti mwana wanu? | | 1. Ku nyumba 2. Ku chipatala | |  |
| 024 | Munalandira chithandizo cha chipatala mutabeleka? | | 1. Eya 2. Ayi | | Ngati ayi pitani funso 034 |
| 025 | Munalandira kangati chithandizo cha chipatala mutabeleka? | | 1. Kamodzi 2. Kawiri mpaka katatu   3. kupitilira katatu | |  |
| 026 | Anakuthandidzani ndani pamene mumalandira chithandizo cha chipatalachi? | | 1. Namwino/Mzamba 2. Dokotala 3. Wophunzira 4. Wothandizira madokolala/anamwino   99. Ena (Nenani)………………………….. | |  |
| 027 | Mumapita kukalandira chithandizo chanji ku sikelelo ya amayi omwe abeleka? (mukhoza kuchonga zopyola chimodzi) | | 1. Chithandizo cha matenda 2. Katemera wa mwana 3. Njira za kulera 4. Kudzapimisa ine ndi mwana   99. Zina (Nenani)…………………………. | |  |
| 028 | Ndizithu ziti zomwe munazikonda ku sikelelo ya amayi omwe abeleka? (mukhoza kuchonga zopyola chimodzi) | | 1. Kosamalilika      1. Ogwira ntchito a nsangara 2. Ndinalandira chithandizo   chokwanira   1. Ndinalandira maphunziro abwino 2. Anandithandidza mwachangu   99. Zina (Nenani)…………………………... | |  |
| 029 | Ndizithu ziti zomwe simunazikonde ku sikelelo ya amayi omwe abeleka? (mukhoza kuchonga zopyola chimodzi) | | 1. Ndinadikira nthawi yaitali 2. Wogwira ntchito anali amwano 3. Ndinathandizidwa ndi wophunzira 4. Samathandiza motsatila nthawi   yomwe munthu wabwelera  99. Zina (Nenani)…………………………... | |  |
| 030 | Anakukopani kuti mukalandire chisamaliro cha chipatala potsatila kubereka kwanu anali ndani? | | 1. Mayi anga 2. Anzanga 3. Woyandikana nawo 4. A mpingo   99. Ena (Nenani)…………………………… | |  |
| 031 | Munachiona bwanji chithandizo chomwe munalandira kumbali ya ubwino wake? | | 1. Chabwino kwambiri 2. Chabwinoko 3. Sichinali bwino 4. Sichinali bwino kwambiri   99. Zina (Nenani)……………………………. | |  |
| 032 | Munawona bwanji kumbali ya khalidwe la ogwira ntchito ku sikelelo ya amayi omwe abeleka? | | 1. A nsangara 2. A mwano 3. Odzikonda   99. Zina (Nenani)………………………… | |  |
| 033 | Munadikilira nthawi yaitali bwanji musanathandizidwe? | | 1. Osapyola ola limodzi 2. Pakati pa ola limodzi ndi awiri 3. Kupyola maola awiri | |  |
| 034 | Chinakupangitsani kuti musadzalandire chisamaliro mutabeleka ndi chani? (mukhoza kuchonga zopyola chimodzi) | | 1. Sindimadziwa za chisamalirochi 2. Ndinali ndi thanzi labwino 3. Ndinatanganidwa 4. Ndimakhala kutali ndi chipatala 5. Ndimaopa kudikira nthawi yaitali   99. Zina (Nenani)……………………….. | |  |
| 035 | Mukuganiza kuti ndi zinthu ziti zomwe ziyenera kukhazikitsidwa kuti tipititse patsogolo kalandiridwe ka chithandizo Pakati pa amayi omwe abeleka m’boma lino la Mangochi? | | 1. Kuchepetsa nthawi yodikilira 2. Ogwira ntchito azipeleka ulemu   kwa wolandira chithandizo   1. Kumayang’anira momwe wophunzira   akugwilira ntchito   1. Kuonjezera ogwira ntchito 2. Kumagwira ntchito ndi nthawi ya nkhomaliro yomwe   99. Zina (Nenani)………………… ……….. | |  |

*Zikomo kwambiri chifukwa chotengapo gawo pa kafukufukuyi*
