# Supplementary material for: Determinants of postnatal care service utilization among mothers of Mangochi district, Malawi: a community-based cross-sectional study
Source: BMC Pregnancy Childbirth. 2021 Aug 30;21:591. doi: 10.1186/s12884-021-04061-4 (PMC8406845; doi:10.1186/s12884-021-04061-4)
Supplement: Supplementary file 3 — Additional file 3: Supplementary File 3. Logistic regression analysis for postnatal care service utilization on socio-demographic and maternal related factors. [file 12884_2021_4061_MOESM3_ESM.docx]

| Variables | PNC Utilization (%) | | COR(CI) | AOR(CI) | P-values |
| --- | --- | --- | --- | --- | --- |
| Age | Yes | No |  |  |  |
| 15-19^R^ | 83.7 | 16.3 | 1.00 | 1.00 | 1.00 |
| 20-24 | 84 | 16 | 1.02 (0.52-2.01) | 0.98 (0.49-1.97) | 0.96 |
| 25-29 | 85 | 15 | 1.08 (0.51-2.28) | 0.92 (0.41-2.03) | 0.83 |
| 30-34 | 83.3 | 16.7 | 0.97 (0.44-2.14) | 0.81 (0.38-1.72) | 0.57 |
| 35-49 | 92.7 | 7.3 | 2.48 (0.76-8.09) | 2.28 (0.71-7.36) | 0.15 |
| Marital status |  |  |  |  |  |
| Single^R^ | 81.4 | 18.6 | 1.00 | 1.00 | 1.00 |
| Married | 85.5 | 14.5 | 1.34 (0.69-2.57) | 1.22 (0.61-2.41) | 0.58 |
| Divorced/Widowed | 82.1 | 17.9 | 1.05 (0.33-3.29) | 1.45 (0.43-4.91) | 0.55 |
| Mother's education level |  |  |  |  |  |
| None^R^ | 75.8 | 24.2 | 1.00 | 1.00 | 1.00 |
| Primary | 86.1 | 13.9 | 1.98(1.19-3.28) | 1.80(1.01-3.19) | 0.005 |
| Secondary & above | 92 | 8 | 3.65(1.49-8.94) | 2.31(1.97-6.04) | 0.001 |
| Partner's education level |  |  |  |  |  |
| None_RC_ | 75.3 | 24.7 | 1.00 | 1.00 | 1.00 |
| Primary | 86.5 | 13.5 | 2.09 (1.09-4.02) | 1.66 (0.88-3.13) | 0.115 |
| Secondary & above | 87.9 | 12.1 | 2.38 (1.28-4.42) | 1.45 (1.25-2.49) | 0.004 |
| Occupation of partner |  |  |  |  |  |
| None^R^ | 78.5 | 21.5 | 1.00 | 1.00 | 1.00 |
| Farmer | 83.3 | 16.7 | 1.37 (0.77-2.44) | 2.38 (1.20-4.72) | 0.039 |
| Business | 91.3 | 8.7 | 2.89 (1.32-6.34) | 3.39 (1.40-8.18) | 0.006 |
| Civil servant | 91.2 | 8.8 | 2.86 (1.14-7.15) | 3.17 (1.25-8.01) | 0.014 |
| Household Income |  |  |  |  |  |
| <K20,000^R^ | 70.4 | 29.6 | 1.00 | 1.00 | 1.00 |
| K20,000-K50,000 | 88.5 | 11.5 | 3.23 (1.66- 6.27) | 2.82 (1.40-5.70) | 0.000 |
| K50,000-K150,000 | 96.6 | 3.4 | 12.06 (5.08-28.65) | 14.41 (5.90-35.16) | 0.000 |
| >K150,000 | 94.4 | 5.6 | 7.14 (2.08-24.54) | 4.63 (1.43-15.03) | 0.000 |
| Decision making |  |  |  |  |  |
| Self^R^ | 77.9 | 22.1 | 1.00 | 1.00 | 1.00 |
| Husband | 87.1 | 12.9 | 1.90 (1.08-3.35) | 1.60 (1.48-2.89) | 0.017 |
| Joint | 85.9 | 14.1 | 1.73 (0.94-3.17) | 2.27 (1.13-4.57) | 0.031 |
| Other | 90.2 | 9.8 | 2.61 (1.22-5.58) | 1.96 (1.85-4.50) | 0.007 |

**Table 5**: Logistic regression analysis of Socio-demographic characteristics associated with PNC utilization of mothers in Mangochi, Malawi (n =600).

**Table 6**: Logistic regression analysis of maternal related factors with PNC of mothers in Mangochi, Malawi (n =600).

| **Variables** | **PNC Utilization (%)** | | **COR(CI)** | **AOR(CI)** | **P-values** |
| --- | --- | --- | --- | --- | --- |
| Parity | Yes | No |  |  |  |
| One^R^ | 84.5 | 15.5 | 1.00 | 1.00 | 1.00 |
| 2--3 | 85.5 | 14.5 | 1.08 (0.63-1.87) | 1.05 (0.59-1.89) | 0.86 |
| 4--5 | 86.4 | 13.6 | 1.16 (0.60-2.25) | 0.95 (0.47-1.90) | 0.88 |
| >5 | 79.3 | 20.7 | 0.70 (0.33-1.51) | 0.85 (0.34-2.10) | 0.73 |
| Ever heard of PNC services |  |  |  |  |  |
| No^R^ | 61.5 | 38.5 | 1.00 | 1.00 | 1.00 |
| Yes | 89 | 11 | 5.06 (3.00-8.56) | 4.06 (2.22-7.41) | 0.000 |
| Heard of postnatal danger signs | |  |  |  |  |
| No^R^ | 57.7 | 42.3 | 1.00 | 1.00 | 1.00 |
| Yes | 89.3 | 10.7 | 6.14 (3.58-10.54) | 4.00 (2.09-7.50) | 0.000 |
|  |  |  |  |  |  |
| Place of delivery |  |  |  |  |  |
| Home^R^ | 46.9 | 53.1 | 1.00 | 1.00 | 1.00 |
| Health facility | 89.4 | 10.6 | 9.52 (5.20-17.44) | 6.88 (3.35-14.14) | 0.000 |

**Note R = Reference category**
