# Supplementary material for: Determinants of postnatal care service utilization among mothers of Mangochi district, Malawi: a community-based cross-sectional study
Source: BMC Pregnancy Childbirth. 2021 Aug 30;21:591. doi: 10.1186/s12884-021-04061-4 (PMC8406845; doi:10.1186/s12884-021-04061-4)
Supplement: Supplementary file 4 — Additional file 4: Supplementary File 4. Results of multicollinearity test for selected predictors for the multivariable model. [file 12884_2021_4061_MOESM4_ESM.docx]

**Table 7**: Results of multicollinearity test for selected predictors for the multivariable model

| Predictors | VIF | Tolerance |
| --- | --- | --- |
| **Socio-demographic characteristics** |  |  |
| Education level of the mother | 1.11 | 0.9 |
| Education level of the partner | 1.08 | 0.93 |
| Occupation status of the mother | 1.05 | 0.96 |
| Occupation status of the partner | 1.03 | 0.97 |
| Household Income |  |  |
| Decision making | 1.04 | 0.96 |
| **Maternal related Characteristics** |  |  |
| Ever heard of PNC Services | 1.22 | 0.82 |
| Heard of Postnatal danger signs | 1.36 | 0.74 |
| Place of delivery | 1.22 | 0.82 |
| Mean VIF | 1.14 |  |
