# Supplementary material for: Determinants of postnatal care service utilization among mothers of Mangochi district, Malawi: a community-based cross-sectional study
Source: BMC Pregnancy Childbirth. 2021 Aug 30;21:591. doi: 10.1186/s12884-021-04061-4 (PMC8406845; doi:10.1186/s12884-021-04061-4)
Supplement: Supplementary file 5 — Additional file 5: Supplementary File 5. Regression diagnostics. [file 12884_2021_4061_MOESM5_ESM.docx]

**Table 8:** Regression diagnostics

| Regression | P-values | AIC |
| --- | --- | --- |
| Logistic regression | 0.000 | 348.23 |
| Complementary log-log | 0.000 | 351.11 |
| Probit | 0.000 | 357.98 |

AIC: Akaike’s Information Criterion
